# Acute Bleeding From Jejunal Malignant Melanoma With an Unusual Similar Lesion in the Right Parotid Gland

**DOI:** 10.1155/cris/4095444

**Published:** 2026-04-18

**Authors:** Shumarova Svetlana, Semkov Anatoli

**Affiliations:** ^1^ Department of Surgery, University Hospital “Aleksandrovska” Sofia, Medical University, Sofia, Bulgaria, mu-sofia.bg; ^2^ Department of Thoracic Surgery, UMHAT St Ivan Rilski, Medical University, Sofia, Bulgaria, mu-sofia.bg

**Keywords:** jejunum, malignant melanoma, melanoma of the parotid gland, small bowel

## Abstract

Metastatic spread of malignant melanoma to the small intestine occurs more frequently than primary intestinal melanoma, which remains an exceptional finding. Clinical manifestations often include gastrointestinal hemorrhage, intestinal obstruction, or acute peritonitis. Prompt surgical intervention is essential in emergency presentations, both to control life‐threatening bleeding and to improve survival prospects. This report describes a rare case of jejunal melanoma presenting with massive gastrointestinal bleeding and an additional tumor with identical histological characteristics in the right parotid gland.

## 1. Introduction

Malignant melanoma arises from the malignant transformation of melanocytes, cells derived from the neural crest during embryonic development. Based on data from the Surveillance, Epidemiology, and End Results (SEER) program, melanoma ranks as the fifth most commonly diagnosed cancer in the United States, with ~106,000 new cases reported in 2021, accounting for about 5.6% of all malignancies [1]. Some authors suggest that, in rare instances, primary melanomas of the small intestine may develop from neuroendocrine Amine Precursor Uptake and Decarboxylation (APUD) cells undergoing neoplastic change or from Schwann‐like cells within the intrinsic autonomic nervous system [2].

Management strategies for melanoma depend largely on disease stage, associated complications, and the overall clinical condition of the patient. In this report, we describe a 71‐year‐old man who presented with acute gastrointestinal bleeding caused by jejunal melanoma and a synchronous lesion with similar histopathological features located in the right parotid gland, making identification of the primary site uncertain.

## 2. Case Presentation

**Figure 1:**
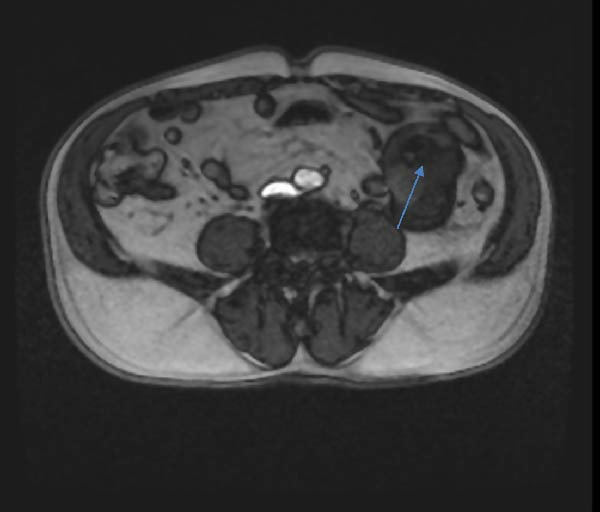
MRI of the small bowel showing a lesion that is mainly T1 hyperintense and T2 hypointense. An irregular segmental wall thickening is noted without proximal dilatation above the stenosis.

**Figure 2:**
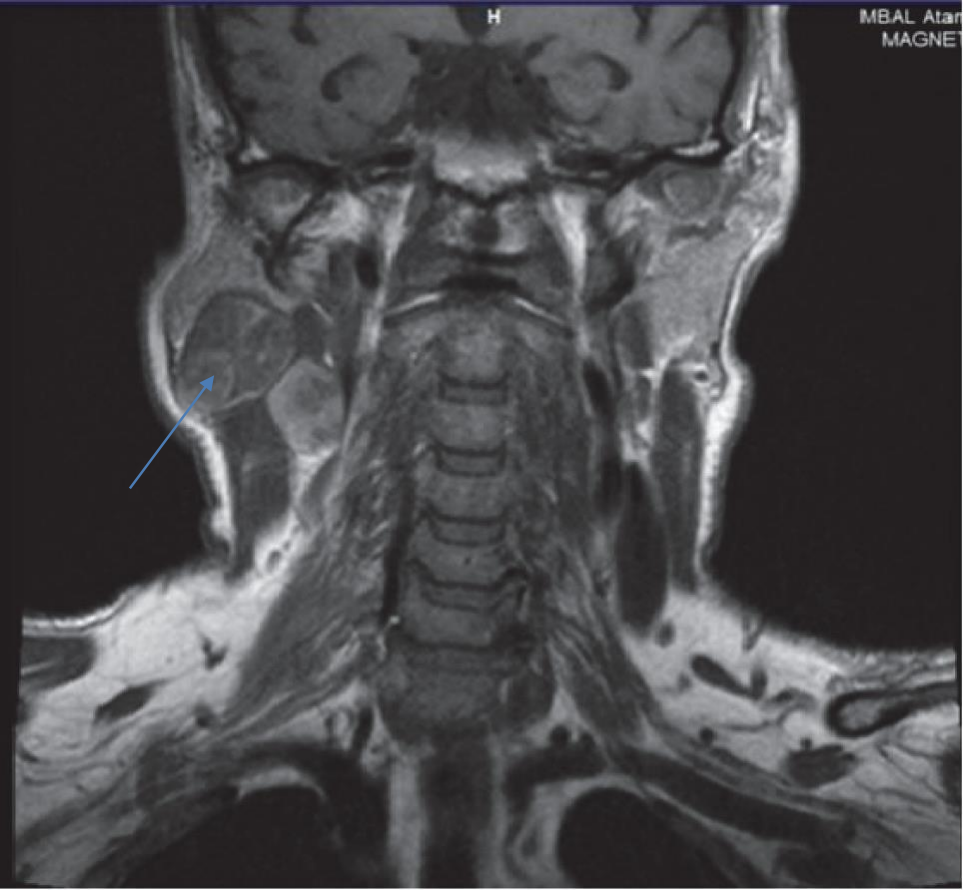
Magnetic resonance imaging showing a right parotid gland mass.

A 71‐year‐old man entered a gastroenterology clinic with complaints of abdominal discomfort, decreased appetite, weight loss, heartburn, and burning behind the sternum. A week before, he noticed a swelling around the area of the right parotid gland. Laboratory results indicated iron‐deficiency anemia, with a hemoglobin (Hb) level of 85 g/L. Tumor markers, including CA 19‐9, CEA and CA 72‐4, were normal. Abdominal ultrasound (US) revealed a tumor formation around the area of the head of the pancreas with dimensions of about 3.6 cm. By a subsequent computed tomography (CT) scan of the abdomen two formations in the head of the pancreas were found out measuring 3.74 cm absorbing the contrast material (34 HU on noncontrast images, increasing to 44 HU in the arterial phase) and an oval formation measuring 4.22 by 6.20 cm involving a small bowel loop with a similar density characteristic without completely obturating the bowel. Magnetic resonance imaging (MRI) showed enlarged lymph nodes along the greater and lesser curvature of the stomach with an inhomogeneous structure, mainly T1 hyperintense, T2 hypointense. Separately, a finding is described in a segment of the small bowel as irregular thickening of the wall, without dilatation аbove the stenosis (Figure 1). A tumor mass was also identified in the right parotid gland (Figure 2). Gastroscopy and colonoscopy were performed, without data on pathological findings. The patient received a blood transfusion and was discharged with an Hb level of 90 g/L.

**Figure 3:**
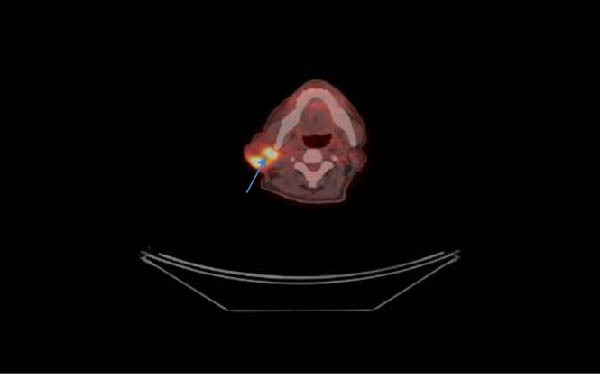
PET/CT scan showing increased metabolic activity in the region of the parotid gland.

**Figure 4:**
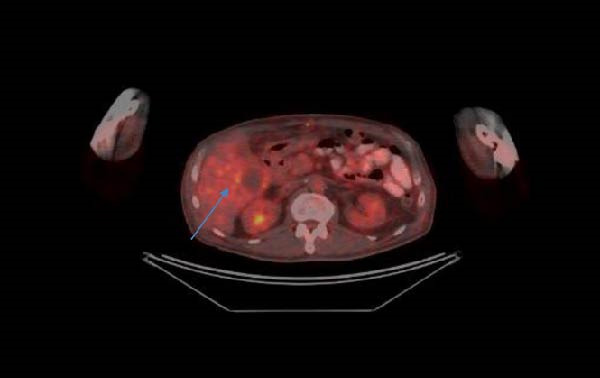
PET/CT data of metabolic activity in the liver.

A biopsy of the tumor in the right parotid gland was performed, revealing a malignant process, though the exact histology could not be determined. Immunohistochemistry (IHC) was negative for TTF‐1, CD 56, p63, CD3, CD 20, Synaptophysin, AE1/AE3. One week after rehospitalization, the patient was admitted to the surgical clinic with melena and a reported Hb level of 75 g/L. Several blood transfusions were administered until stabilization of the patient’s condition. A median laparotomy was performed, revealing a jejunal tumor measuring ~6 cm, causing obstruction of the bowel lumen, with an easily bleeding surface and dilated loops proximal to the lesion. Intraoperatively, no liver lesions were observed. Small bowel resection with primary anastomosis was performed. Histopathological examination revealed pigmented malignant melanoma. Following stabilization of the patient’s condition, restaging with positron emission tomography/CT (PET/CT) was performed. This revealed increased metabolic activity in the region of the parotid gland, SUVmax 8.0 (Figure 3), as well as lower‐grade activity in the regions of the pancreatic head and the liver (Figure 4), without a clearly identifiable primary melanoma focus. The patient was consulted with a dermatologist, and no skin lesions suspicious for melanoma were found and had no previous cancer or immunosuppressive treatment. Further IHC analysis demonstrated HMB‐45 positivity, S‐100 positivity, and a Ki‐67 proliferation index of 70%. The findings were consistent with pigmented malignant melanoma with high mitotic activity. No BRAF gene mutation was detected. Immunotherapy was initiated; however, the patient survived no longer than 9 months following surgery, most likely due to the patient’s comorbidities.

## 3. Discussion

Epidemiological data on the gender distribution of malignant melanoma remain inconsistent. Sung et al. [3] reported age‐standardized incidence rates of 3.8 per 100,000 for men and 3.0 per 100,000 women, corresponding to lifetime risks of 0.42% and 0.33%, respectively. In a large histopatological review of 1105 biopsy‐proven gastrointestinal melanomas, Shah et al. [4] identified small‐bowel involvement in 4.7% of cases. According to a previous study [2], melanomas originating on the extremities (15%–57%), followed by those of the trunk (13%–54%) and the head and neck (5%–33%), are most likely to metastasize to the gastrointestinal tract. A primary melanoma of the parotid gland cannot be excluded, despite its extreme rarity [5]. Our patient had neither a prior history of cutaneous melanoma nor any evidence of such lesions on thorough physical examination, making the jejunal tumor and concurrent parotid lesion diagnostically challenging. The difficulty in determining the primary site is further complicated by the presence of multiple metabolically active lesions in other parts of the body at the time of diagnosis, which does not exclude secondary involvement of the parotid gland, as also reported by other authors [6].

Melanoma of the small intestine, whether primary or metastatic, often remains clinically silent until complications develop. Depending on the site and extent of the disease, patients may experience diverse symptoms, including abdominal pain [7–13], gastrointestinal bleeding [8, 10, 13, 14], vomiting due to intestinal obstruction [9, 13], or even acute peritonitis [11] (Table 1). The diagnosis pathway is largely guided by the clinical presentation. Abdominal ultrasonography is typically the first imaging modality performed, while gastroscopy and colonoscopy are used in cases presenting with melena, although they frequently fail to identify the bleeding source. When small‐bowel involvement is suspected, capsule endoscopy or enteroscopy can provide valuable visualization of mucosal lesions. Cross‐sectional imaging modalities such as contrast‐enhanced CT and MRI are commonly employed [7–17]; however, PET/CT remains the most reliable tool for staging and detection of multifocal disease. Immunohistochemical markers, including S‐100, Melan‐A, and HMB‐45, are highly sensitive for confirming the melanocytic origin of such tumors [7, 10–12] (Table 1).

**Table 1:** Data extracted from clinical cases.

No. of patients	Reference	Journal	Year	Age (years)	Gender	Clinical presentation	Imaging	Site	Complications	First localization	S100	MelanA	HMB45	Operation	Exitus
1	Chen [8]	Cureus	2024	31	Male	Pain	CT	Ileum	Intussusception	Melanoma of the scalp	Positive	Positive	Posotive	Laparoscopic	No
2	Froes [14]	Annals of Medicine and Surgery	2022	64	Male	Melena	CT	Small bowell	Bleeding	Melanoma of the right shoulder	—	—	—	Robitic	No
3	Gracas [8]	Frontiers in Surgery	2022	55	Male	Pain, melena	CT, MRI	Jeunum	Bleeding	—	Positive	Positive	—	Laparoscopic	No
4	Sa [9]	JSCR	2021	82	Male	Pain, vomiting	CT	Jeunum	Intussusception	Melanoma in the right preauricular	Positive	—	Posotive	Laparotomy	No
5	Pacilli [15]	Int J Surg Case Rep	2023	58	Male	Abdominal swelling	CT, MRI	Ileum	Intestinal perforation	Melanoma of the right conjunctiva	—	—	—	Laparotomy	No
6	Vilar [16]	Medicine Internationale	2024	47	Male	—	CT, MRI	Ileum	Intussusception	—	Positive	Positive	Posotive	Laparoscopic	No
7	Marak [10]	Radilol Case Rep	2024	58	Male	Pain, melena	CT	Ileum	Bleeding	—	Positive	Positive	Posotive	Biopsia	Yes
8	Khooei [11]	Iranian Journal of Phatology	2024	42	Male	Pain, vomiting	X‐ray	Ileum	Peritonitis	—	Positive	Positive	Posotive	Laparotomia	No
9	Mattit [12]	Int J Surg Case Rep	2024	52	Male	Pain	CT	Jeunum	Obstruction	No	Positive	Positive	Posotive	Laparoscopic	No
10	Wu [13]	JSCR	2022	71	Male	Pain, vomiting, melena	CT	Ileum	Obstruction, bleeding	No	Positive	Positive	—	Laparoscopic	No
11	Kharroub [17]	Int J Surg Case Rep	2022	48	Female	Diarrhea	CT, PET CT	Ileum	Intussusception	Melanoma of the left heel	—	—	—	Laparoscopic	No

The role of surgery in metastatic malignant melanoma has changed substantially with the advent of effective systemic therapies [2]. Despite this, many patients with small bowel involvement still present acutely with intestinal obstruction, hemorrhage, or peritonitis, circumstances that necessitate prompt surgical management by experienced teams. Prognosis in metastatic melanoma is influenced by multiple factors, including disease stage, mutational profile, and overall patient condition. Among genetic alterations, the BRAF V600 mutation has received particular attention as a target for molecular therapy. It occurs in roughly 40%–50% of melanoma cases and responds to BRAF inhibitors‐specifically antibodies targeting CTLA‐4 and PD‐1‐has also significantly improved outcomes for advanced melanoma, producing objective response rates between 20% and 40% [18].

## 4. Conclusion

Despite the generally poor prognosis in metastatic melanomas, complex treatment by both surgical methods and targeted therapy, as well as immunotherapy, is necessary to increase overall survival. Unfortunately, in emergency situations of metastatic small bowel melanoma, such as ileus, bleeding, surgical treatment, although palliative, is a necessary lifesaving option.

## Author Contributions

Svetlana Shumarova performed the surgical procedure as the primary surgeon, collected the patient’s clinical and laboratory data, and wrote the original version of the manuscript. Semkov Anatoli reviewed the manuscript.

## Funding

The author received no specific funding for this work.

## Consent

Written informed consent was obtained from the patient’s relatives for publication of this case report and all accompanying images.

## Conflicts of Interest

The authors declare no conflicts of interest.

## Data Availability

The data that support the findings of this study are available from the corresponding author upon reasonable request.
